# Late onset Alzheimer’s disease: modeling disease hallmarks via in vitro 3D iNeuron cultures

**DOI:** 10.1038/s41392-024-01999-7

**Published:** 2024-10-10

**Authors:** Giacomo Masserdotti

**Affiliations:** 1 Institute of Stem Cell Research, Helmholtz Center Munich, 85764 Neuherberg, Germany; 2 Biomedical Center Munich (BMC), Physiological Genomics, LMU Munich, 82152 Planegg-Martinsried, Germany

**Keywords:** Regeneration and repair in the nervous system, Diseases of the nervous system

A study recently published in *Science*^1^ reported a novel in vitro model system for Alzheimer’s disease (AD), based on the direct neuronal reprogramming of human fibroblasts from control and AD patients, that successfully replicates several hallmarks of the disease. As such, it further advances in vitro modeling of AD, allowing not only to investigate the molecular mechanisms behind it, but also to perform high-throughput screenings aimed at identifying potential therapeutic treatments.

AD is the most common cause of dementia, a neurodegenerative disease characterized by progressive memory loss and thinking impairment.^2^ The further decline of these symptoms often leads to behavioral and social inability. Post-mortem analysis of AD brains shows the extracellular accumulation of amyloid-β (Aβ) plagues, insoluble tau formation containing a mixture of 3R- and 4R-tau isoforms, neurite degeneration, and irreversible neuronal loss. Over the years, several AD-causing genes with autosomal dominance (ADAD) have been identified, but they represent only 1–2% of AD cases. Among the risk factors associated with AD, aging is certainly the most relevant: most AD cases occur after age 65, therefore termed late-onset AD (LOAD), while early-onset AD (EOAD) constitutes only 5% of AD patients. Thus, developing a reliable model system for AD should consider age as an essential parameter. Different animal models have been developed to mimic ADAD but cannot model LOAD—simply because of the difference in lifespan to human beings; likewise, iPSC-derived AD neurons cannot model aging, as iPSC generation resets the age signature of the donor cells to embryonic age.

Direct neuronal reprogramming is a promising avenue to generate neurons from differentiated, non-neuronal cell types: this is achieved via the forced expression of reprogramming factors.^3^ Notably, direct neuronal reprogramming does not erase the aging signature: therefore, induced neurons (iNs) retain the age of the cells targeted for the conversion.^4^ However, when fibroblasts from AD patients were reprogrammed in 2D cultures via the forced co-expression of Ngn2 and Ascl1, the resulting iNs exhibited age-associated dysregulation, but no AD hallmarks, thus suggesting that the culture system or the reprogramming factors were not sufficient to mimic AD pathology.

Sun and colleagues built upon these observations and developed a new in vitro model system with two major differences: the reprogramming cocktail and the culture conditions. In fact, they expressed miRNA124/miR-9/9* together with NEUROD2 and MYT1L in human fibroblasts to generate cortical iNs^5^ and, instead of maintaining them in 2D, the authors embedded the cells in either a low-density thin gel made of 15% Matrigel (3D-CNs), or high-density spheroids. Following 2–3 weeks of reprogramming, fibroblasts converted efficiently into cortical iNs, as shown via immunofluorescent analysis and RNA sequencing. Most importantly, the protocol was successfully tested in fibroblasts from ADAD (mutations in *PSEN1* or *APP*; 4 lines), LOAD (6 lines) patients, and 17 age- and sex-matched healthy controls, thus allowing to investigate the molecular mechanisms, the influence of age of the starter cell and the differences between ADAD and LOAD iNs.

First, the authors evaluated the presence of AD hallmarks, such as extracellular Aβ plagues deposition, phosphorylated-tau, K63-ubiquitin, and the co-expression of both 3R- and 4R-tau isoforms, in ADAD-iNs and compared them to healthy controls. Remarkably, ADAD-iNs, either cultured in 3D-CNs or spheroids, showed all these AD signs. Furthermore, the early and prolonged treatment with β- or ɣ-secretase inhibitors reduced Aβ deposition, thus showing the possibility of modeling successful treatments. ADAD-iNs contained also seed-competent tau, as assessed by FRET analysis. Moreover, they were also transcriptionally different from healthy control counterparts, expressing higher levels of genes associated with increased AD risk, such as *MMP3*, *IBSP*, and *TAGLN4*. Importantly, 3D-CNs ADAD-iNs showed neurite degeneration over time, as well as cell death, thus recapitulating neurodegeneration.

**Fig. 1:**
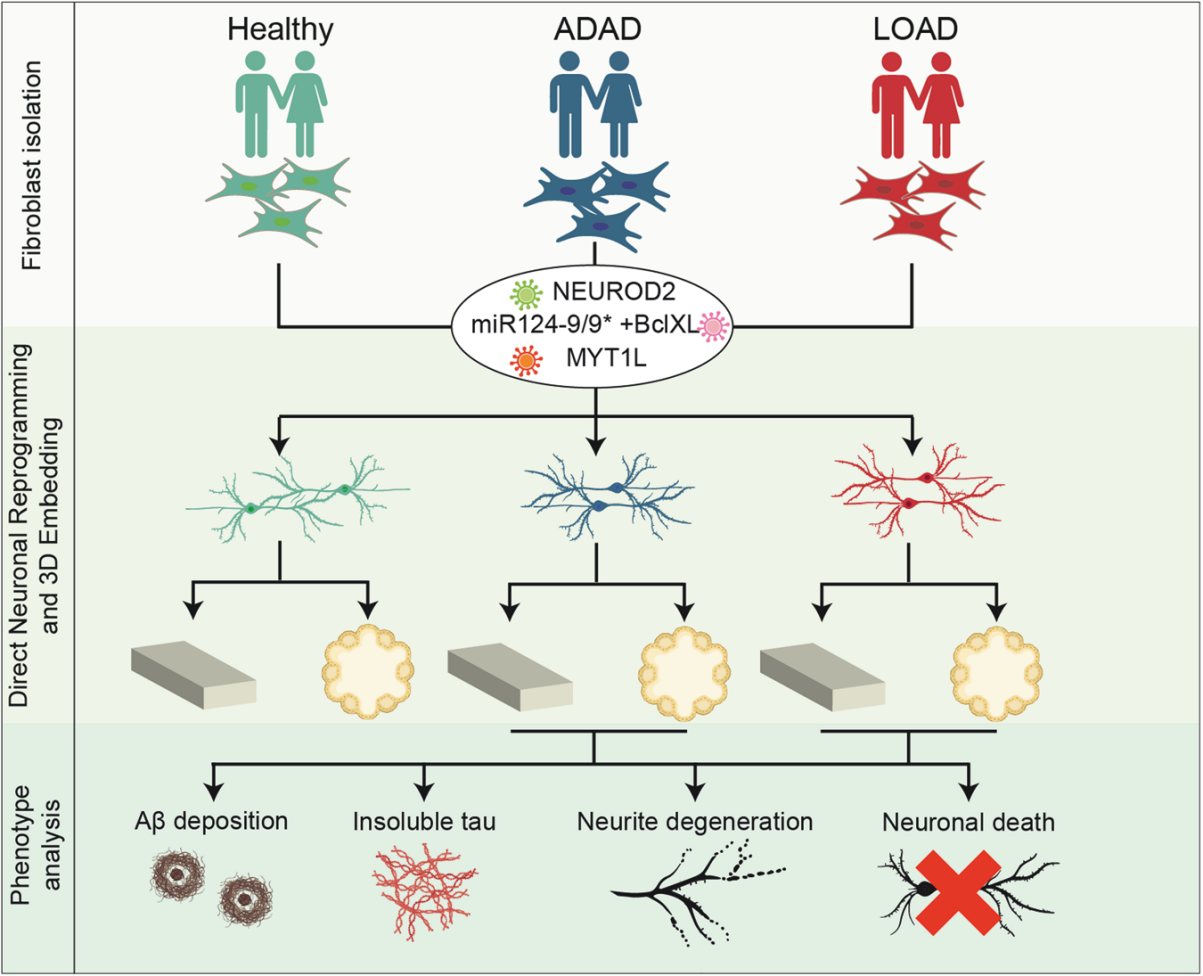
Graphical summary of the findings by Sun et al.^1^: cortical-like neurons, generated by direct neuronal conversion of fibroblasts from healthy donors or patients suffering from ADAD or LOAD, show key hallmarks of AD observed in patients. The figure was created with BioRender.com and Adobe Illustrator CS6

Then, the authors examined if these phenotypes may also be detectable in LOAD samples. Indeed, 3D-CNs or spheroids LOAD-iNs showed Aβ plagues deposition, tau pathology, impairment of synapse formation, neurite degeneration, and cell death over time. Likewise, LOAD-iNs had a different transcriptome than controls, highlighting the higher expression of metallopeptidases and genes associated with the inflammatory response. The comparison with published datasets from postmortem human prefrontal cortex from AD individuals unraveled several genes elevated in LOAD-iNs that are associated with aging, inflammatory response, and apoptosis, further supporting the reliability of the in vitro model (Fig. 1).

As tauopathy has been recently associated to the dysregulation of retrotransposon elements (RTE), the authors evaluated RTE expression and found that its increase correlates with age rather than with the pathology. Early treatment with lamivudine (3TC), an inhibitor of reverse transcriptase, robustly decreased the presence of single-strand DNA (ssDNA) — an intermediate product of retrotransposition used as an indicator for RTE synthesis — reducing also neuronal death and Aβ formation. This was associated to reduced DNA damage and to the increased expression of genes associated with immune and inflammatory response, possibly indicating that lamivudine suppresses neurodegeneration in LOAD-iNs by regulating RTE-mediated neuroinflammation. Remarkably, a similar treatment in ADAD-iNs had no effect, highlighting differences in the two types of AD groups.

The authors also examined the relationship between Aβ deposition and neurodegeneration in LOAD-iNs by treating cells with ɣ-secretase inhibitors. Interestingly, treatment at the onset of Aβ deposition (16 days post induction, PID) but not a later time point (PID22) substantially reduced Aβ plague formation, lowering phosphorylated-tau and neuronal death, thus suggesting that ɣ-secretase inhibition is not effective in reducing or delaying the phenotype once the Aβ plagues are formed.

The work of Sun and colleagues represents a significant advancement of in vitro cultures as a disease modeling system for complex brain disorders like AD. Despite the limited time of observation (30 PID), this model provides a powerful platform to explore the pathophysiology of AD across different clinical categories (genetic, early-onset, or late-onset), and in future to investigate the role of other cell types (e.g., astrocytes, oligodendrocytes, and microglia) in AD-related degeneration. On the other hand, developing a 96-well plate platform enhances the scalability of this model, making it a valuable tool for high-throughput screening aimed at identifying small molecules or genes that could prevent early Aβ deposition or more crucially, halt the further spread of tau pathology.
